# *Salix* transect of Europe: variation in ploidy and genome size in willow-associated common nettle, *Urtica dioica* L. *sens. lat.*, from Greece to arctic Norway

**DOI:** 10.3897/BDJ.4.e10003

**Published:** 2016-09-27

**Authors:** Quentin Cronk, Oriane Hidalgo, Jaume Pellicer, Diana Percy, Ilia J. Leitch

**Affiliations:** ‡ University of British Columbia, Vancouver, Canada; § Royal Botanic Gardens, Kew, United Kingdom; | Natural History Museum, London, United Kingdom

**Keywords:** megatransect, genome size, cytotype variation, *
Urtica
*

## Abstract

**Background:**

The common stinging nettle, *Urtica dioica* L. sensu lato, is an invertebrate "superhost", its clonal patches maintaining large populations of insects and molluscs. It is extremely widespread in Europe and highly variable, and two ploidy levels (diploid and tetraploid) are known. However, geographical patterns in cytotype variation require further study.

**New information:**

We assembled a collection of nettles in conjunction with a transect of Europe from the Aegean to Arctic Norway (primarily conducted to examine the diversity of *Salix* and *Salix*-associated insects). Using flow cytometry to measure genome size, our sample of 29 plants reveals 5 diploids and 24 tetraploids. Two diploids were found in SE Europe (Bulgaria and Romania) and three diploids in S. Finland. More detailed cytotype surveys in these regions are suggested. The tetraploid genome size (2C value) varied between accessions from 2.36 to 2.59 pg. The diploids varied from 1.31 to 1.35 pg per 2C nucleus, equivalent to a haploid genome size of c. 650 Mbp. Within the tetraploids, we find that the most northerly samples (from N. Finland and arctic Norway) have a generally higher genome size. This is possibly indicative of a distinct population in this region.

## Introduction

During a recent study of willow (*Salix* spp.) stands on a latitudinal transect across Europe (Cronk et al. 2015) the opportunity arose to sample individuals of Urtica dioica L. ssp. dioica (the common stinging nettle) that frequently co-occurs with willow in riparian habitats (see under Materials and Methods for further details). *Urtica dioica* is one of the most remarkable plants of Europe. First it possesses a defense, stinging hairs, which are a small marvel of biochemistry and biomechanics. These are highly effective against vertebrate herbivores (Levin 1973, Pollard and Briggs 1984, Pullin and Gilbert 1989, Tuberville et al. 1996). The cell walls of the trichome tip are silicified and brittle (Haberlandt 1914, Barber and Shone 1966, Thurston and Lersten 1969, Thurston 1974, Sowers and Thurston 1979) and break off (like the tip of a glass ampoule) on the slightest mechanical stimulation. The fluid released is a potent and complex mixture of toxins including histamine, oxalic acid and tartaric acid (Emmelin and Feldberg 1949, Fu et al. 2006, Taskila et al. 2000).

Secondly it has an extraordinary biogeographical range, occurring in every corner of Europe, from the shores of the Mediterranean to the Arctic Ocean and from the winter-cold central European plain to the rainswept coasts of western Ireland. Few plants have the ability to grow in such a wide range of climatic conditions. Over this range it is largely native, having spread along its natural habitat of rich alluvial river floodplains. However, it has also become an aggressive ruderal, taking advantage of human disturbance to complete its conquest of Europe through accidental introduction by humans.

Thirdly it is an invertebrate “super-host”. Throughout Europe it provides the food plant for large numbers of specialist and generalist insects, notably in the Lepidoptera, Coleoptera and Hemiptera (Davis 1973, Davis 1975, Davis 1983, Davis 1989, Perrin 1975).

Fourthly, it has exceptional mineral nutrition, being highly phosphate demanding. It ceases growth if phosphate is limiting and responds luxuriantly if phosphate is added, whereas in contrast plants adapted to poor soil scarcely respond to additional phosphate (Pigott 1971, Pigott and Taylor 1964, Taylor 2009). It is not only an indicator of high available phosphate, but it is also a general mineral accumulator, having high concentrations of calcium, nitrogen and phosphorus in its tissues (Müllerová et al. 2014). This may go some way to explaining its attractiveness to herbivorous invertebrates.

Taxonomically *Urtica dioica* is part of a complex of closely related taxa and subtaxa (Grosse-Veldmann and Weigend 2015), which includes U. dioica subsp. subinermis (R. Uechtr.) Hand & Buttler, U. dioica subsp. sondenii (Simmons) Hyl. and U. dioica subsp. pubescens (Ledeb.) Domin (Table 1). In addition there are a number of related European perennial nettles that are sometimes confused with *Urtica dioica*, although they are distinctive. These include: *Urtica gracilis* Aiton (the American stinging nettle), *Urtica kioviensis* Rogow. and *Urtica membranacea* Poir. (Table 1; nomenclature follows Euro+Med PlantBase 2006). Most of these taxa are diploid (Table 1) (typically 2n=26) except for U. dioica subsp. dioica (common nettle), which is reported as largely, but not completely, tetraploid (Table 1).

Two types of cytological diversity have been found in Urtica dioica subsp. dioica. One is the reported difference in tetraploid chromosome number between 2n=48 and 2n=52 (Skalińska et al. 1974). Such a discrepancy could be due to miscounts, but the repeated reports of both numbers leads to a suspicion that both numbers do exist in nature.

There is also the difference in ploidy level. The possibility must be entertained that counts for *Urtica dioica* of 2n=26 (diploid) refer to one of the infraspecifc taxa and not to U. dioica subsp. dioica. However there are numerous counts that are candidates for genuine diploid U. dioica subsp. dioica. For instance Kolnik and Goliašová (in Mráz 2006), reported a chromosome count of 2n=26 for *Urtica dioica* from Závod, Slovakia. Because of the problematic taxonomy of this group it is very important that herbarium voucher specimens are collected in conjunction with any study.

Genome size estimates have also been made for *Urtica dioica* (see Bennett and Leitch 2012 and additional data not yet incorporated into the database), and these results (Table 2) are also indicative of cytotype diversity. Nevertheless, the same cautionary taxonomic considerations apply as well as technological issues arising from the estimation of genome size (e.g. Doležel et al. 2007, Greilhuber et al. 2007, Pellicer and Leitch 2014).

## Materials and Methods

*Context of study*

*Urtica dioica* samples (Table 3) were collected during a survey of willow habitats in a latitudinal transect across Europe: the *Salix* transect of Europe (Cronk et al. 2015). The aim of this was to survey variation in *Urtica* and one of its constant herbivores, the *Urtica* psyllid, *Trioza urticae*, which were co-sampled. Information on *Trioza* will be the given in separate papers. Herbarium and living *Urtica* samples were collected. The aim of the current work was to investigate the extent of ploidy level and genome diversity within the resultant *Urtica* collection.

*Site selection and sampling*

**Figure 1.**
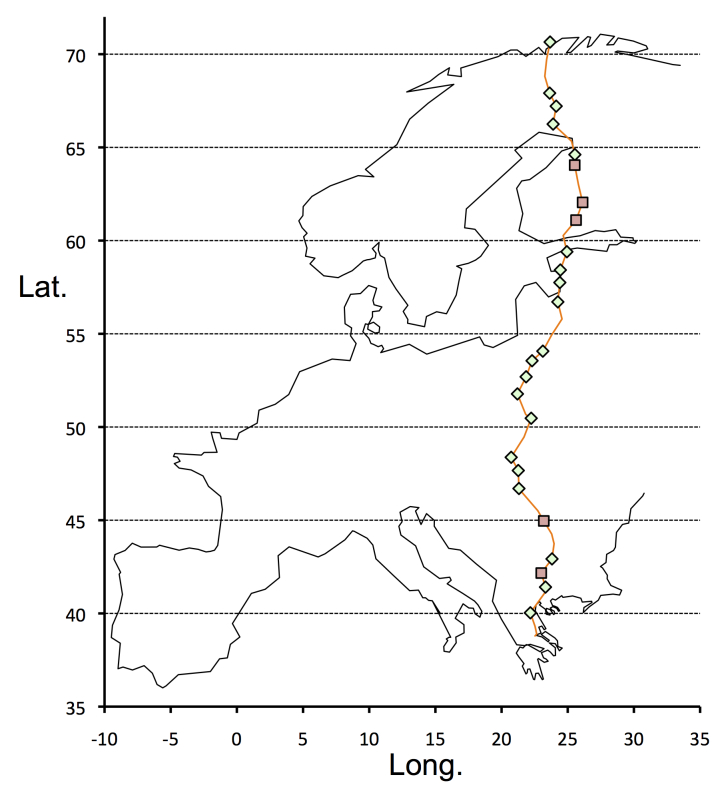
Map of *Urtica* sample sites. Squares: diploids; diamonds: tetraploids; red line = route of transect (Lat. = latitude, Long. = longitude).

Full details of the sites (mainly riverine alluvial habitats), and their selection are given in Cronk et al. (2015). The sites are summarized in Table 3. In all, 42 *Salix* sites were chosen between Athens (Greece) and Hammerfest (Norway) (Fig. 1). Of these 33 (and one supplementary site) had *Urtica dioica* present and a herbarium voucher specimen was collected from each of these sites (and in addition one specimen of *U. membranacea* from Greece). Herbarium voucher specimens are deposited in the herbarium of the Natural History Museum, London (BM). Living specimens were also collected for cultivation in London (Queen Mary University of London), for future experimental work. One living specimen was collected from each site (two from site 27). Of the living specimens collected, 27 survived into cultivation and could be used for flow cytometry (see results). The living specimens were grown in London in a 'common garden' (rooftop plant growth facility at Queen Mary University of London, Lat. 51.5234. Long. -0.0423).

*Flow cytometry and buffers*

Ploidy level (diploid vs tetraploid) was assayed using flow cytometry (as described in Hanson 2005), using a Partec CyFlow flow cytometer with *Petroselinum crispum* (parsley) 'Champion Moss Curled' 2C=4.50 pg (Obermayer 2002) as calibration standard. A range of different flow cytometry buffers were tested (including the Galbraith buffer, the general purpose buffer and the LB01 buffer, Pellicer and Leitch 2014). However, only the ‘CyStain PI Absolute P kit’ buffer (Sysmex UK) gave acceptable flow histograms with CVs routinely less than 3%, so it was chosen for estimating the ploidy level of the 29 *Urtica* specimens.

## Results

The flow cytometry results are given in Table 3. In all, 24 plants have flow cytometry results consistent with tetraploidy whereas five plants, 7-5 (Bulgaria), 11-4 (Romania), while 31-12, 32-11, 34-6 (southern Finland), have results consistent with diploidy. The identity of these diploid plants was checked and confirmed as *U. dioica* *sens. lat.* As diploidy is often associated with stinglessness, information on the presence of stinging hairs was collected after cultivation in a common garden (Queen Mary University of London, QMUL) for one year (Table 4). Information on flowering time in the common garden is also given. Flowering time shows an overall correlation with latitude (generally with late flowering plants coming from Finland and Norway, although there are some exceptions (Table 4). Voucher specimens of both the original specimens and plants after cultivation (in common garden conditions for one year) are deposited at the Natural History Museum, London (BM).

**Figure 2.**
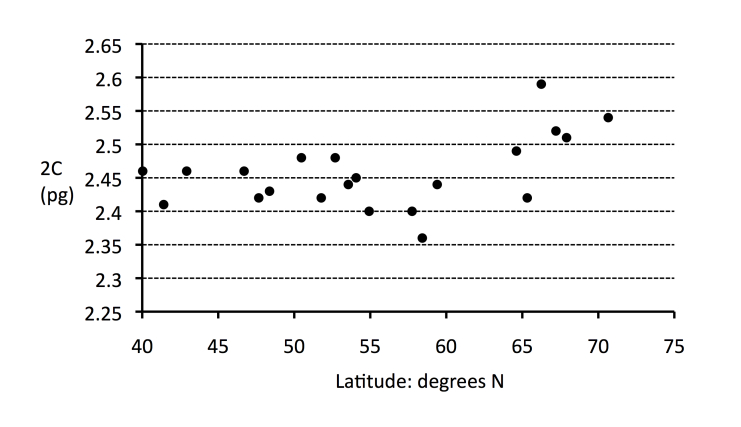
Scatter plot of genome size (2C-value, pg) values (as given in Table 3), plotted against latitude (Table 3). Only tetraploids (4x) are shown; diploid samples (2x) are not plotted. Note the generally higher genome size of the high latitude samples (see Table 3).

**Figure 3.**
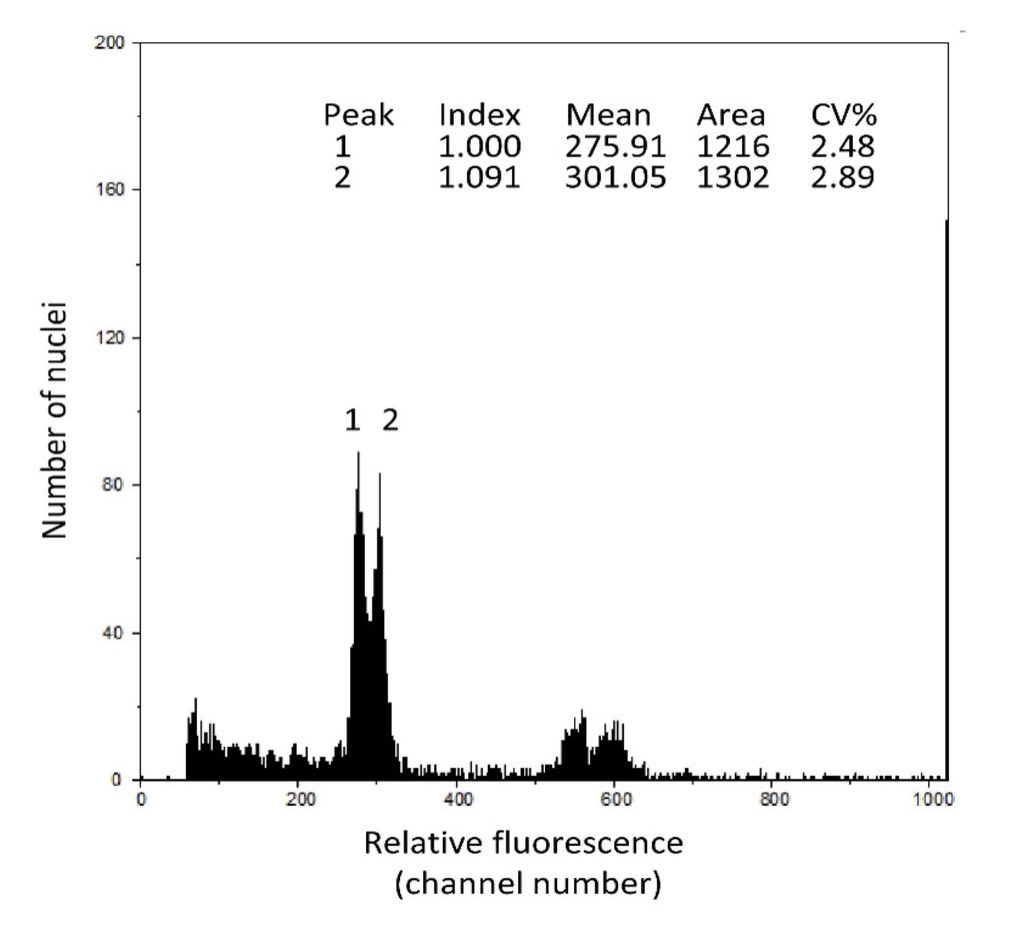
Screen shot from the Partec CyFlow flow cytometer showing flow histogram obtained from analysing *Urtica dioica* accessions 28-10 (peak 1, 2C=2.36 pg) and 37-6 (peak 2, 2C=2.59 pg) showing two distinct peaks and hence demonstrating genuine intraspecific variation in genome size between these two tetraploid individuals (28-10 and 37-6: see Table 3). The graph shows the relative fluorescence (indicative of DNA amount) in thousands of cell nuclei. The machine also gives summary statistics for the peaks. Note the very low coefficient of variation (CV%) of 2.48% and 2.89%.

At the tetraploid level, some variation in the estimated genome sizes was detected, with the northern populations tending to have higher 2C-values compared with the more southerly ones (Table 3; Fig. 2). To confirm that this intraspecific variation was genuine rather a technical artefact, leaves from the two individuals showing the largest difference in 2C-value (i.e. 28-10, 37-6) were co-processed. This resulted in two distinct peaks in the flow histogram (Fig. 3), indicative of biologically real difference in C-values at the tetraploid level.

## Discussion

The results confirm that the tetraploid is the dominant cytotype in our sample of *U. dioica* but that diploid plants do occur relatively frequently (at least in SE Europe and S. Finland). A more extensive survey of cytotype variation in Romania and Bulgaria, as well as around the Baltic would be of interest. Ploidy level has been shown to correspond with morphological characters (Geltman 1984; Geltman 1986). The possibility must be therefore be examined that the diploid samples here belong to the diploid taxa Urtica dioica ssp. pubescens (Ledeb.) Domin (synonym: U. dioica ssp. galeopsifolia (Wierzb. ex Opiz) J. Chrtek), U. dioica ssp. subinermis (R. Uechtr.) Weigend or Urtica dioica ssp. sondenii (Simmons) Hyl. These taxa typically lack stinging hairs on the leaves. U. dioica ssp. pubescens typically has a pubescence of long non-stinging hairs whereas U. dioica ssp. sondenii is glabrous. We are cautious in assigning any of the individuals studied here to those taxa without further study of the populations, which may not be homogeneous. Of the diploids, only 11-4 and 32-11 can be considered stingless. None of the diploids here are glabrous (although many have an indumentum of very short hairs), ruling out U. dioica ssp. sondenii. Only 32-11 (with few stinging hairs on leaves and relatively long pubescence) can be considered a reasonable match for U. dioica ssp. pubescens. However this plant generally resembles the other diploids, 31-12 and 34-6, which vary in stinging hairs and pubescence. Until the populations from which these plants come can be examined critically we tentatively assign all our samples to the variable U. dioica ssp. dioica.

The finding of diploids in SE Europe raises the possibility that the widespread tetraploid form of Urtica dioica subsp. dioica, which has also become a weed, may have originated there, and the diploids may have survived glacial episodes in S. European refugia. The origin of the diploids of S. Finland is as yet unknown, although a phylogeographic analysis might be informative here. Another interesting result is the discovery of intraspecific C-value variation, particularly the generally higher C-values in the far north. This may be indicative of a distinct population of nettles in the north, and again this would benefit from more detailed cytogeographic study.

## Figures and Tables

**Table 1.** Some nettle taxa reported in Europe with representative chromosome counts. There are very large numbers of counts for *Urtica dioica* and the list below does not aim to be comprehensive. For a full summary see the Chromosome Counts Database, CCDB (Rice et al. 2014).

**Name**	**Notes**	**Representative chromosome counts**
U. *dioica* L. subsp. *dioica*	The common stinging nettle	2n=26 (Kolník M. and Goliašová, in Mráz 2006); 2n=48 (Májovský et al. 1987); 2n=48, 52 (Skalińska et al. 1974); 2n=48, 52 (Lippert 2006); 2n=52 (Löve and Kjellqvist 1974); 2n=52 (Corsi et al. 1999)
			U. dioica subsp. subinermis (R. Uechtr.) Weigend		2n=24/26 (Lippert 2006)
U. dioica subsp. sondenii (Simmons) Hyl.		2n=26 (Geltman 1984)
U. dioica subsp. pubescens (Ledeb.) Domin	Syn. *U. galeopsifolia*	2n=26 (Geltman 1984); 2n=26 (McAllister 1999)
*U. gracilis* Aiton	Syn. U. dioica subsp. gracilis (Aiton) Selander	2n=26, 52 (Woodland et al. 1982)
*U. kioviensis* Rogow.		2n=26 (Kolník M. and Goliašová, in Mráz 2006)
*U. membranacea* Poir.		2n=22 (Corsi et al. 1999)

**Table 2.** Previous genome size estimates in *Urtica dioica* s.l. *Fe = Feulgen microdensitometry, FC:PI = Flow cytometry using propidium iodide

**Taxon name given in study**	**2C-value (pg)**	**Chromosome number if available (2n)**	**Origin of material**	Comment*	**Reference**
*U. dioica*	1.22	n/a	Canada	Estimated using FC:PI with LB01 or MgSO_4_ buffer and Solanum lycopersicum L. ‘Stupické polní rané’ (2C=1.96 pg) as calibration standard.	Bainard et al. 2011
*U. dioica*	3.1	52	UK	Estimated using Fe with Senecio vulgaris (PBI population (2C=3.16 pg) as calibration standard.	Mowforth 1986
*U. dioica*	2.34	n/a	Germany	Estimated using FC:PI with Galbraith buffer. Calibration standard unclear.	Barow and Meister 2003
*U. dioica*	2.16	n/a	West Balkans, Central Bosnia, Serbia Macedonia	Estimated using FC:PI with Galbraith buffer and Petunia hybrid ‘PxPC6’ (2C=2.85 pg) as calibration standard.	Pustahija et al. 2013

**Table 3.** Locations of the *Urtica* samples collected in April and June 2015, together with estimated genome size (2C-values) and ploidy levels made from the living material (herb. = only herbarium material available).

**Sample**	**Latitude (N)**	**Longitude (E)**	**Country**	**River/ location**	**2C-value (pg)**	**Ploidy Level (x)**	**Living material/Flow cytometry**
2-4	38.902	22.31015	Greece	R. Sperchios, near Leianokladi, east of Lamia	-	-	Herb. only
4-4	40.032685	22.175437	Greece	Stream near Kokkinogeia, Thrace	2.46	4	Yes
5-3	41.113317	23.273893	Greece	At R. Struma, near Lithotopos	-	-	Herb. only
6-5	41.412468	23.318609	Bulgaria	R. Struma, near Topolnitsa	2.41	4	Yes
7-5	42.165622	22.998141	Bulgaria	R. Struma, north of Boboshevo	1.35	2	Yes
8-3	42.923989	23.810563	Bulgaria	R. Kalnitza, near Botevgrad	2.46	4	Yes
11-4	44.961981	23.190337	Romania	R. Jiu, north of Rovinari	1.33	2	Yes
12-3	45.510676	22.737225	Romania	Meadow near Paucinesti, Carpathian region	-	-	Herb. only
13-4	46.518504	21.512839	Romania	R. Crisul Alb, at Chisineu-Cris	-	-	Herb. only
14-6	46.700744	21.31268	Hungary	R. Fekete-Koros, near Gyula	2.46/ 2.46	4	Yes (x2)
15-5	47.665648	21.261768	Hungary	Drainage ditches near R. Hortobagy, north-east of Balmazujvaros	2.42	4	Yes
16-7	48.374291	20.725264	Hungary	R. Bodva, south of Szendro	2.43	4	Yes
17-4	49.463447	21.697255	Poland	R. Panna, at Tylawa	-	-	Herb. only
18-4	50.470234	22.238372	Poland	Fields north of Rudnik nad Sanem	2.48	4	Yes
19-7	50.673994	21.823391	Poland	R. Leg, near Gorzyce	-	-	Herb. only
20-6	51.775039	21.1971	Poland	R. Pilica, at Warka	2.42	4	Yes
21-11a	52.69398	21.8529	Poland	R. Bug, near Brok	2.48	4	Yes
22-6	53.55483	22.30299	Poland	Meadow near R. Biebrza at Wasocz, near Szczuczyn	2.44	4	Yes
23-6	54.06943	23.11745	Poland	R. Czarna Hancza, near Sejny on road from Suwalki	2.45	4	Yes
24-11	54.92583	23.7742	Lithuania	Embankment of River at Kaunas	2.40	4	Yes
26-15	56.71141	24.25162	Latvia	Near R. Misa, between Iecava and Kekana	-	-	Herb. only
27-6 & 7	57.74963	24.4023	Latvia	R. Salaca short distance inland from Salacgriva	2.40	4	Yes (27-7)
28-10	58.42257	24.44063	Estonia	Field near Parnu	2.36	4	Yes
29-7	59.40289	24.93577	Estonia	R. Pirita at Lagedi near Tallinn	2.44	4	Yes
30-8	60.27299	24.65843	Finland	Near Lake Bodom, Espoo, Finland	n.d.	n.d.	Yes
31-12	61.09965	25.6282	Finland	Drainage flowing into lake Vesijärvi at Paimela near Lahti	1.33	2	Yes
32-11	62.04962	26.12369	Finland	Lake near Toivakka	1.34	2	Yes
34-6	64.05074	25.52664	Finland	R. Pyhäjoki, at Joutenniva, south of Haapavesi	1.31	2	Yes
35-8	64.61287	25.53805	Finland	Tributary of the R. Siikajoki near Mankila	2.49	4	Yes
37-6	66.24947	23.8945	Finland	Small river between Kainuunkylä and Väystäjä	2.59	4	Yes
38-11	67.21253	24.12629	Finland	Near Vaattojärvi	2.52	4	Yes
39-16	67.91183	23.63411	Finland	River Muonion (Muonionjoki) just south of Muonio	2.51	4	Yes
42-8	70.65234	23.66583	Norway	Jansvannet Lake, Hammerfest	2.54/ 2.53	4	Yes (x2)
SUPPLEMENTARY SITES							
i-D-1 & 2	38.1261	22.45348	Greece	[*Urtica membranacea*]	-	-	Herb. only (fem. & mas.)
ii-D-4	65.32443	25.3153	Finland	Kestilä	2.42	4	Yes

**Table 4.** *Urtica* phenotype in common garden (London). Fl. (flowering) time refers to category of flowering performance in 2016; 1 = early flowering (flowering before 16 May); 2 = mid-June (flowering by 10 June); 3 = late June (21 June); 4 = early July (2 July); 5 = late or not flowering (not flowering by early July). Stinging hairs refers to the typical number of stinging hairs per leaf; 1 = <10; 2 = 10-50; 3 = 50-100; 4 = >100. Numbers are given for: adaxial surface (first number)/abaxial surface (second number).

**Accession**	**Fl. time**	**Stinging hairs**	**Notes**
4-4	1	3/3	Well-armed.
6-5	2	2/3	Moderately well-armed.
7-5 (diploid)	4	1/3	Tall plant with rather narrow leaves but abundant stinging hairs on undersides of leaves. Non-stinging hairs very short.
8-3	5	1/3	Moderately well-armed.
11-4 (diploid)	3	1/1	Leaves largely stingless except on petiole. Shortly pubescent on veins and stems.
14-6	1	3/4	Well-armed.
15-5	2	1/1	Leaves largely stingless, except on petiole. Shortly pubescent on veins.
16-7	2	3/4	Well-armed.
18-4	2	3.4	Well-armed.
20-6	2	3/4	Well-armed.
21-11	2	2/3	Moderately well-armed.
22-6	3	3/4	Well-armed.
23-6	2	3/4	Well-armed.
24-11	2	1/1	Largely stingless except on petiole and midrib. Pubescent on veins.
27-7	3	3/4	Well-armed.
28-10	3	1/1	Largely stingless except on petiole, pubescent with rather long hairs on veins.
29-7	2	3/4	Well-armed.
30-8	5	2/3	Moderately well-armed.
31-12 (diploid)	3	1/3	Moderately armed below, other pubescence of rather sparse very short hairs.
32-11 (diploid)	3	1/1	Leaves very largely unarmed below, stinging hairs mainly on inflorescence, petiole and stem, otherwise similar to previous, but stems and veins covered with longer non-stinging hairs.
34-6 (diploid)	4	1/3	Moderately well-armed; other pubescence of very short hairs.
35-8	3	3/4	Well-armed.
37-6	5	2/3	Moderately well-armed.
38-11	4	2/3	Moderately well-armed.
39-16	5	2/3	Moderately well-armed.
42-8	3	3/4	Well-armed.
42-8	4	3/4	Well-armed.
FIN-D4	5	3/4	Well-armed.
